# Longitudinal Trajectories of Estimated Glomerular Filtration Rate in a European Population of Living Kidney Donors

**DOI:** 10.3389/ti.2024.13356

**Published:** 2024-08-26

**Authors:** Manuela Almeida, Pedro Reis Pereira, José Silvano, Catarina Ribeiro, Sofia Pedroso, Sandra Tafulo, La Salete Martins, Miguel Silva Ramos, Jorge Malheiro

**Affiliations:** ^1^ Department of Nephrology, Unidade Local de Saúde de Santo António (ULSdSA), Porto, Portugal; ^2^ UMIB - Unit for Multidisciplinary Research in Biomedicine, ICBAS - School of Medicine and Biomedical Sciences, University of Porto, Porto, Portugal; ^3^ ITR - Laboratory for Integrative and Translational Research in Population Health, Porto, Portugal; ^4^ Instituto Português do Sangue e da Transplantação, Porto, Portugal; ^5^ Department of Urology, Unidade Local de Saúde de Santo António (ULSdSA), Porto, Portugal

**Keywords:** kidney transplant, living donor, estimated glomerular filtration rate, living donor characteristics, estimated glomerular filtration rate trajectories

## Abstract

A living donor (LD) kidney transplant is the best treatment for kidney failure, but LDs safety is paramount. We sought to evaluate our LDs cohort’s longitudinal changes in estimated glomerular filtration rate (eGFR). We retrospectively studied 320 LDs submitted to nephrectomy between 1998 and 2020. The primary outcome was the eGFR change until 15 years (y) post-donation. Subgroup analysis considered distinct donor characteristics and kidney function reduction rate (%KFRR) post-donation [−(eGFR_6 months(M)_–eGFR_pre-donation_)/eGFR_pre-donation_*100]. Donors had a mean age of 47.3 ± 10.5 years, 71% female. Overall, LDs presented an average eGFR change 6 M onward of +0.35 mL/min/1.73 m^2^/year. The period with the highest increase was 6 M–2 Y, with a mean eGFR change of +0.85L/min/1.73 m^2^/year. Recovery plateaued at 10 years. Normal weight donors presented significantly better recovery of eGFR +0.59 mL/min/1.73 m^2^/year, compared to obese donors −0.18L/min/1.73 m^2^/year (*p* = 0.020). Noteworthy, these results only hold for the first 5 years. The subgroup with a lower KFRR (<26.2%) had a significantly higher decrease in eGFR overall of −0.21 mL/min/1.73 m^2^/year compared to the groups with higher KFRR (*p* < 0.001). These differences only hold for 6 M–2 Y. Moreover, an eGFR<50 mL/min/1.73 m^2^ was a rare event, with ≤5% prevalence in the 2–15 Y span, correlating with eGFR pre-donation. Our data show that eGFR recovery is significant and may last until 10 years post-donation. However, some subgroups presented more ominous kidney function trajectories.

## Graphical Abstract

**Figure:**
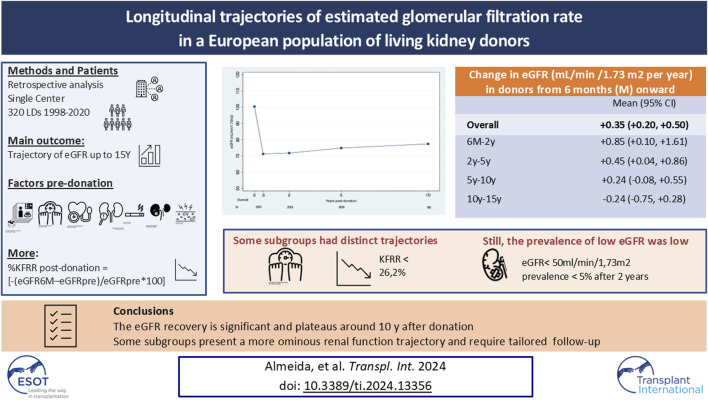

## Introduction

A living donor (LD) kidney transplant (KT) is the best treatment for end-stage renal disease (ESRD) [1–3]. LDKT increases organ availability, decreases time on the waiting list, allows preemptive transplantation, and improves graft and patient survival with lower healthcare costs [1–5]. Although the perceived risks for the donors are considered low and ethically acceptable [3], two landmark studies showed an increased risk of ESRD in donors compared with matched healthy non-donors, albeit the absolute risk was minimal [6, 7]. Subgroups with a higher risk of ESRD have been identified [8, 9], but post-donation kidney function evolution and the mechanisms involved in the hyperfiltration after donation are less well characterized [10–14]. Furthermore, due to organ scarcity, we are increasingly accepting donors with borderline abnormalities that were previously declined [15]. Long-term follow-up data are scarce.

For guiding clinical practice, it would be desirable to foresee the evolution of kidney function after nephrectomy in each LD and the meaningful identification of markers that could identify individuals at higher risk in whom preventive measures of chronic kidney disease (CKD) and ESRD [16] could be sought more stringently. The 2017 Kidney Disease Improving Global Outcomes (KDIGO) Workgroup published extensive clinical practice guidelines for evaluating LD candidates [16]. They recommend that transplant programs provide each candidate with individualized quantitative estimates of short-term and long-term risks from the donation and a personalized follow-up plan [16]. However, the document does not provide precise instructions on how to do that.

We sought to retrospectively describe the estimated glomerular filtration rate (eGFR) change in our cohort of LDs and evaluate if it changes differently according to baseline donor characteristics and the kidney function reduction rate (KFRR) 6 months post-donation. We also investigated the prevalence of low eGFR (<50 mL/min/1.73 m^2^) in different donor subgroups and proteinuria after donation. We hypothesize that identifying different patterns of eGFR change after donation could signal groups of LDs that would benefit from a better risk assessment and customized preventive care.

## Patients and Methods

We retrospectively reviewed the clinical data of all adult LDs submitted to nephrectomy at our center between January 1998 and January 2020 (n = 364). Inclusion criteria were serum creatinine (Scr) evaluation at 6 months and at least 3 Scr evaluations at follow-up (31 LKDs without Scr evaluations at 6 months and 13 without at least 3 evaluations at follow-up were excluded from the analysis, further details of non-included donors are available as Supplementary Material). The remaining 320 LKDs defined our studied cohort.

The Institutional Review Board at Unidade Local de Saúde de Santo António (ULSdSA) approved this retrospective observational study, which was conducted according to the Helsinki Declaration.

### Donor Variables

Following international guidelines [16, 17], all donors were subjected to a standard evaluation protocol. Baseline demographic, anthropometric, analytical, and clinical data were collected from the LDs. Hypertension was defined by blood pressure in the consultation >140/90 mmHg, ABPM >135/85 mmHg, and past hypertension or antihypertensive medication. Uncontrolled hypertension or evidence of end-organ damage were criteria of exclusion. Potential donors with a history of malignancy, obesity, or diabetes were excluded. Serum creatinine-based CKD-Epidemiology Collaboration (CKD-EPI equation) [18] was used to predict eGFR. Although a lower limit of eGFR was not established by Unit protocol, potential donors with eGFR below 80 mL/min/1.73 m^2^ were usually discarded. Upon urinary analysis, proteinuria was defined by a random urine protein/creatinine ratio of 0.15–0.5 g/g [19] and was confirmed by determination using a 24-h urinary sample. Donors with confirmed proteinuria over 300 mg/day were discarded [20, 21]. The final approval for kidney donation was reviewed in a multidisciplinary meeting, and ethical approval was mandatory.

The date of nephrectomy was defined as the beginning of follow-up. All donors have lifetime annual follow-up appointments.

### Outcomes

The primary outcome was the change in eGFR until 15 years post-donation (ml/min/1.73m^2^/per year), using all available eGFR measurements from 6 months after the nephrectomy onward. Donors were followed from the nephrectomy date until one of the following occurred: death, ESRD, attaining 15-year follow-up, or end-of-study period (31 December 2022). We performed additional analyses to examine the effects of various characteristics on the progression of eGFR over time in living kidney donors presenting at the time of donation that could be associated with the recovery of kidney function after donation [22–25], including demographic and clinical data. Further, the kidney function reduction rate (%KFRR) post-donation [−(eGFR_6months(M)post-donation_–eGFR_pre-donation_)/eGFR_pre-donation_*100] in the remnant kidney in the first 6 months after donation was considered in the analysis, bearing in mind it was a variable available only after donation.

### Statistical Analysis

Continuous data were described using mean and standard deviation (SD) or median (interquartile range [IQR]), and categorical data were expressed as numbers (and percentages). Categorical data were compared using Pearson chi-square test or Fisher exact test, and continuous variables were compared with Student’s t-test or Mann–Whitney U-test.

Subgroup analysis considered the following donor characteristics: age, sex, obesity, diagnosis of hypertension, smoking habits, proteinuria, and pre-donation eGFR category; these same variables were included in the multivariable model. Differently, %KFRR post-donation as an independent predictor was evaluated separately, adjusted to the aforementioned donor characteristics pre-donation.

Donor eGFR change between 6 months and 15 years post-donation was assessed by univariate and multivariable linear mixed regression model that imputed subject-specific random effects (intercept and slope defined as eGFR at 6-month and time in years, respectively) on an unstructured covariance matrix. The Bonferroni test was used to correct multiple significance tests. The dependent variable was all eGFR measurements, and the independent variables were entered as 2-way interaction terms between them and the time (in years) variable. Additionally, distinct temporal trends of eGFR change were sought by imputing time as a linear spline with knots at 2, 5, and 10 years.

Statistical calculations were performed using STATA/MP, version 15.1 (Stata Corp, College Station, TX, United States). A 2-sided *P*-value <0.05 was considered as statistically significant.

## Results

### Baseline Characteristics

The baseline characteristics for our study cohort are summarized in Table 1. The mean age of the population was 47.3 ± 10.5 years, and most were female (71%). The representation in the race of donors was nearly exclusively Caucasian. Most donors were either overweight (41%) or obese (10%). Fifteen percent had smoking habits, 14% had dyslipidemia, and 16% had hypertension. Pre-donation mean eGFR was 100.4 ± 14.6 mL/min/1.73 m^2^. 76% of the cohort had eGFR >90 mL/min/1.73 m^2^, and 29 donors (9%) had eGFR <80 mL/min/1.73 m^2^. Ninety-six donors (30%) had proteinuria.

**TABLE 1:** Baseline characteristics of the study cohort.

	n = 320
Age (years), Mean ± SD	47.3 ± 10.5
Age (years), n (%) <40 40–55 ≥55	81 (25) 154 (48) 85 (27)
Sex F:M, n (%)	227 (71):93(29)
BMI kg/m^2^, Mean ± SD	25.3 ± 3.3
BMI kg/m^2^, n (%) <25 25–30 ≥30	155 (48) 132 (41) 33 (10)
Smoking habits, n (%)	48 (15)
Hypertension, n (%)	51 (16)
Dyslipidemia, n (%)	44 (14)
ProtU 0.15–0.5 g/g, n (%)	96 (30)
Pre-donation SCr mg/dL, Mean ± SD	0.75 ± 0.16
Pre- donation eGFR mL/min/1.73 m^2^, Mean ± SD	100.4 ± 14.6
Pre- donation eGFR mL/min/1.73 m^2^, n (%) <80 80–90 ≥90	29 (9) 48 (15) 243 (76)
Number of SCr measurements, Median (IQR) [min. max.]	7 (5–11) [3.16]
% kidney function reduction rate (FKRR) post-donation*, Median (IQR)	31.9 (22.6–38.1)
% KFRR post-donation, n (%) <26.2 26.2–36.1 >36.1	106 (33) 107 (33) 107 (33)

*KFRR post-donation = [−(eGFR6M-eGFRpre-donation)/eGFRpre-donation*100].

SD, standard deviation; n, number; F, female: M, male; BMI, Body Mass Index; ProtU, protein/creatinine ratio in the urine; ProtU 0.15–0.5 g/g, a ratio protein/creatinine of 0.15–0.5 g/g in a urinary sample; SCr, Serum creatinine; eGFR, estimated glomerular filtration rate; IQR, interquartile range; KFRR, kidney function reduction rate.

At follow-up, after discharge, the donors had a median number of SCr measurements of 7 (IQR 5–11), performed, per protocol, at 6 months, 1 year after donation, and then yearly.

### Evolution of Renal Function After Donation

The median percentage of KFRR was 31.9 (IQR 22.6–38.1)%, One-third of the cohort had a reduction rate of less than 26.2%, one-third between 26.2% and 36.1%, and one-third greater than 36.1% (Table 1).

**FIGURE 1:**
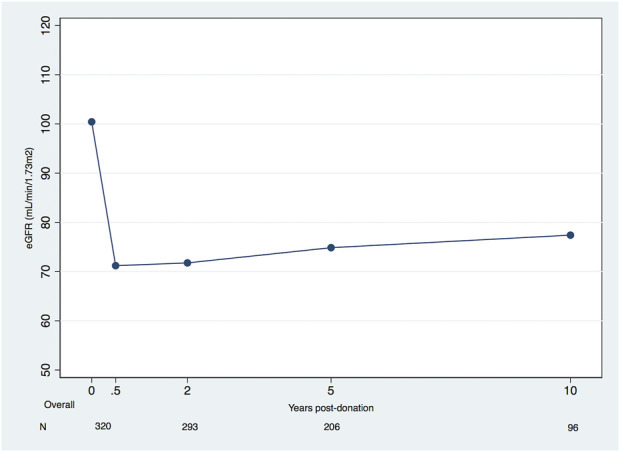
Mean eGFR (mL/min/1.73 m^2^) in living kidney donors pre-donation and during post-donation follow-up (eGFR, estimated glomerular filtration rate).

Overall, after the first 6 months, our cohort’s eGFR increased, on average, +0.35 mL/min/1.73 m^2^/year (95% confidence interval (CI), +0.20 to +0.50). Using the linear spline model results, the average changes of eGFR were, respectively, from 6 months to 2 years, 2–5 years, 5–10 years, and 10–15 years, +0.85 mL/min/1.73 m^2^/year (95% CI, +0.10 to +1.61), +0.45L/min/1.73 m^2^/year (95% CI, +0.04 to +0.86), +0.24 mL/min/1.73 m^2^/year (95% CI, −0.08 to +0.55) and −0.24 mL/min/1.73 m^2^/year (95%CI, −0.75 to +0.28) (Table 2). A plateau was achieved around 10 years (Figure 1).

**TABLE 2:** Change in eGFR (mL/min/1.73 m^2^/year) in 320 donors from 6 months onward.

	Mean (95% CI)
Overall	+0.35 (+0.20, +0.50)
Linear spline model 6M–2y 2y–5y 5y–10y 10y–15y	+0.85 (+0.10, +1.61) +0.45 (+0.04, +0.86) +0.24 (−0.08, +0.55) −0.24 (−0.75, +0.28)

CI, confidence interval; M, months; y, years; eGFR, estimated glomerular filtration rate.

### Donor Subgroup Analysis

Overall, there were no significant differences in the evolution of renal function after donation based on donor characteristics such as age, sex, hypertension, dyslipidemia, and presence of proteinuria (Tables 3, 4).

**TABLE 3:** Changes in eGFR (mL/min/1.73 m^2^/year) in living kidney donors (n = 320) by subgroup over different periods during follow-up from 6 weeks onward (univariate analysis).

		Linear spline model			
	Overall	6M–2y	2y–5y	5y–10y	10–15y
Age (years) <40 40–55 ≥ 55 *p*	+0.39 (+0.11, +0.68)^A^ +0.34 (+0.13, +0.55)^A^ +0.52 (+0.21, +0.82)^A^ *0.642*	+0.09 (−1.44, 1.62)^A^ +1.03 (−0.05, +2.11)^A^ +1.39 (−0.07, +2.84)^A^ *0.457*	+0.97 (+0.14, +1.80)^A^ +0.36 (−0.22, +0.95)^A^ +0.33 (−0.46, +1.13)^A^ *0.445*	+0.10 (−0.51, 0.72)^A^ +0.30 (−0.14, +0.74)^A^ +0.47 (−0.20, +1.14)^A^ *0.73*	−0.04 (−0.94, +0.87)^A^ −0.41 (−1.14, +0.32)^A^ +0.12 (−1.08, +1.32)^A^ *0.698*
Sex Male Female *p*	+0.54 (+0.25, +0.82) +0.28 (+0.10, +0.45) *0.124*	+0.08 (−1.34, +1.50) +1.16 (+0.27, +2.04) *0.209*	+0.79 (+0.00, +1.58) +0.34 (−0.14, +0.82) *0.335*	+0.36 (−0.25, +0.98) +0.20 (−0.17, +0.56) *0.653*	+0.86 (−0.14, +1.85) −0.65 (−1.24, −0.05) ***0.011***
BMI (kg/m^2^)* <25 25–30 ≥30 *p*	+0.50 (+0.28, +0.71)^A^ +0.32 (+0.10, +0.54)^A^ −0.01 (−0.50, +0.47)^A^ *0.143*	+0.46 (−0.62, +1.54)^A^ +2.23 (+1.06, +3.41)^B^ −2.52 (−4.82, −0.22)^C^ ***0.001***	+0.96 (+0.37, +1.54)^A^ −0.29 (−0.92, +0.34)^B^ +1.28 (+0.02, +2.55)^A^ ***0.007***	+0.17 (−0.30, +0.64)^A^ +0.50 (+0.05, +0.96)^A^ −0.51 (−1.60, +0.58)^A^ *0.205*	+0.01 (−0.85, +0.86)^A^ −0.49 (−1.20, +0.22)^A^ +0.08 (−1.44, +1.60)^A^ *0.616*
Hypertension No Yes *p*	+0.33 (+0.17, +0.49) +0.53 (+0.13, +0.93) *0.365*	+0.95 (+0.12, +1.77) +0.42 (−1.47, +2.31) *0.616*	+0.35 (−0.10, +0.79) +1.05 (+0.03, +2.08) *0.215*	+0.30 (−0.04, +0.64) −0.09 (−0.97, +0.79) *0.413*	−0.37 (−0.91, +0.18) +0.83 (−0.69, +2.35) *0.148*
Smoking habits No Yes *p*	+0.32 (+0.15, +0.48) +0.58 (+0.19, +0.97) *0.213*	+1.26 (+0.45, +2.08) −1.52 (−3.48, +0.43) ***0.01***	+0.22 (−0.23, +0.66) +1.92 (+0.84, +3.00) ***0.004***	+0.28 (−0.05, +0.62) +0.02 (−0.84, +0.87) *0.567*	−0.44 (−1.00, +0.13) +0.65 (−0.57, +1.87) *0.114*
Dyslipidemia No Yes *p*	+0.33 (+0.17, +0.49) +0.50 (+0.08, +0.92) *0.459*	+0.82 (+0.00, +1.63) +1.20 (−0.79, +3.20) *0.724*	+0.46 (+0.02, +0.91) +0.41 (−0.70, +1.52) *0.928*	+0.22 (−0.12, +0.55) +0.42 (−0.49, +1.32) *0.682*	−0.31 (−0.87, +0.24) +0.21 (−1.22, +1.63) *0.504*
ProtU 0.15–0.5 g/g No Yes *p*	+0.32 (+0.15, +0.49) +0.44 (+0.14, 0.75) *0.502*	+1.20 (+0.30, +2.11) +0.06 (−1.31, +1.42) *0.17*	+0.22 (−0.26, +0.71) +1.01 (+0.25, +1.77) *0.086*	+0.30 (−0.05, +0.65) +0.05 (−0.67, +0.77) *0.541*	−0.26 (−0.83, +0.30) −0.17 (−1.42, +1.09) *0.891*
Pre-donationeGFR*(mL/min/1.73 m^2^) <80 80–90 ≥90 *p*	−0.06 (−0.61, +0.48)^A^ +0.47 (+0.10, +0.85)^A^ +0.38 (+0.21, +0.55)^A^ *0.256*	+2.84 (+0.31, +5.37)^A^ +2.15 (+0.24, +4.06)^A^ +0.37 (−0.49, +1.24)^A^ *0.07*	−0.06 (−1.58, +1.46)^A^ −0.43 (−1.48, +0.62)^A^ +0.70 (+0.24, +1.16)^A^ *0.122*	−0.88 (−2.08, +0.32)^A^ +0.48 (−0.32, +1.27)^A^ +0.32 (−0.03, +0.68)^A^ *0.146*	−0.75 (−2.23, +0.73)^A^ +1.38 (+0.15, +2.61)^B^ −0.54 (−1.15, +0.07)^A^ ***0.018***
%KFRR post-donation*, ** <26.2 26.2–36.1 >36.1 *p*	−0.12 (−0.34, +0.10)^B^ +0.62 (+0.36, +0.87)^A^ +0.75 (+0.48, +1.02)^A^ ***<0.001***	−2.89 (−4.19, −1.59)^A^ +1.54 (+0.24, +2.83)^B^ +3.77 (+2.50, +5.04)^C^ ***<0.001***	+0.59 (−0.08, +1.26)^A^ +0.88 (+0.17, +1.59)^A^ +0.05 (−0.66, +0.76)^A^ *0.262*	−0.01 (−0.48, +0.46)^A^ +0.25 (−0.30, +0.80)^A^ +0.61 (+0.02, +1.20)^A^ *0.273*	−0.24 (−0.90, +0.43)^A^ −0.29 (−1.36, +0.79)^A^ −0.56 (−1.67, +0.55)^A^ *0.885*

*In variables with 3 or more groups, each box will present letters A to C (superscript). It should be concluded that subgroups that share the same letters in the same box are non-significantly different.

**KFRR post-donation = [−(eGFR6M-eGFRpre-donation)/eGFRpre-donation*100].

y, years; M, months; BMI, body mass index; ProtU, protein/creatinine ratio in the urine; eGFR, estimated glomerular filtration rate; KFRR, kidney function reduction rate.

*p* values are depicted in italics; statistically significant *p* values (<.05) are shown in bold.

**TABLE 4:** Changes in eGFR (mL/min/1.73 m^2^/year) in living kidney donors (n = 320) by subgroup over different periods during follow-up from 6 months onward (multivariable analysis).

		Linear spline model			
	Overall	6mo–2y	2y–5y	5y–10y	10–15y
Age* (years) <40 40–55 ≥55 *p*	+0.36 (+0.07, +0.65)^A^ +0.41 (+0.21, +0.62)^A^ +0.44 (+0.12, +0.75)^A^ *0.932*	+0.66 (−0.94, 2.26)^A^ +1.18 (+0.08, +2.27)^A^ +0.96 (−0.59, +2.51)^A^ *0.87*	+0.69 (−0.18, +1.56)^A^ +0.42 (−0.17, +1.01)^A^ +0.45 (−0.39, +1.30)^A^ *0.879*	+0.05 (−0.61, +0.71)^A^ +0.36 (+0.08, +0.81)^A^ +0.40 (−0.34, +1.14)^A^ *0.706*	+0.08 (−1.03, +1.18)^A^ −0.47 (−1.31, +0.38)^A^ +0.07 (−1.51, +1.36)^A^ *0.727*
Sex Male Female *p*	+0.61 (+0.31, +0.91) +0.33 (+0.16, +0.50) *0.128*	+0.57 (−0.91, +2.05) +1.15 (+0.24, +2.06) *0.522*	+0.60 (−0.23, +1.43) +0.46 (−0.03, +0.95) *0.787*	+0.68 (−0.01, +1.37) +0.15 (−0.25, +0.55) *0.222*	+0.53 (−0.93, +2.00) −0.52 (−1.24, +0.21) *0.242*
BMI* (kg/m^2^) <25 25–30 ≥30 *p*	+0.59 (+0.37, +0.80)^B^ +0.35 (−0.14, +0.56) ^AB^ −0.18 (−0.68, +0.31)^A^ ***0.02***	+0.67 (−0.44, +1.77) ^AB^ +2.17 (+0.98, +3.36)^B^ −2.47 (−4.92, −0.03)^A^ ***0.002***	+0.97 (+0.36, +1.57)^B^ −0.17 (−0.81, +0.46)^A^ +1.18 (−0.17, +2.53) ^AB^ ***0.021***	+0.32 (−0.17, +0.81)^A^ +0.49 (+0.02, +0.95)^A^ −0.64 (−1.82, +0.54)^A^ *0.214*	−0.11 (−1.14, +0.91)^A^ −0.26 (−1.01, +0.48)^A^ −0.62 (−2.39, +1.16)^A^ *0.897*
Hypertension No Yes *p*	+0.37 (+0.21, +0.53) +0.60 (+0.19, +1.01) *0.313*	+1.07 (+0.23, +1.90) +0.58 (−1.48, +2.64) *0.672*	+0.34 (−0.11, +0.80) +1.36 (+0.23, +2.48) *0.11*	+0.37 (+0.01, +0.73) −0.12 (−1.08, +0.83) *0.361*	−0.22 (−0.88, +0.44) −0.29 (−2.17, +1.59) *0.946*
Smoking No Yes *p*	+0.41 (+0.25, +0.56) +0.40 (−0.01, +0.82) *0.985*	+1.31 (+0.48, +2.14) −0.86 (−2.93, +1.20) *0.059*	+0.30 (−0.15, +0.75) +1.66 (+0.48, +2.83) ***0.039***	+0.40 (+0.04, +0.75) −0.31 (−1.32, +0.70) *0.211*	−0.30 (−1.01, +0.42) +0.16 (−1.47, +1.78) *0.648*
Dyslipidemia No Yes *p*	+0.37 (+0.22, +0.53) +0.61 (+0.19, +1.04) *0.311*	+0.92 (+0.10, +1.75) +1.45 (−0.72, +3.62) *0.661*	+0.51 (+0.06, +0.95) +0.43 (−0.78, +1.64) *0.904*	+0.27 (−0.08, +0.62) +0.46 (−0.52, +1.43) *0.725*	−0.30 (−0.96, +0.36) +0.24 (−1.54, +2.03) *0.585*
ProtU 0.15–0.5 g/g No Yes *p*	+0.39 (+0.23, +0.55) +0.46 (+0.16, 0.76) *0.674*	+1.28 (+0.38, +2.20) +0.19 (−1.19, +1.56) *0.193*	+0.32 (−0.16, +0.81) +0.99 (+0.22, +1.75) *0.152*	+0.37 (+0.01, +0.72) +0.08 (−0.65, +0.82) *0.504*	−0.24 (−0.88, +0.41) −0.21 (−1.61, +1.18) *0.975*
Predonation eGFR*(mL/min/1.73m^2^) <80 80–90 ≥90 *p*	−0.09 (−0.63, +0.44)^A^ +0.52 (+0.16, +0.88)^A^ +0.43 (+0.27, +0.60)^A^ *0.141*	+2.85 (+0.24, +5.46)^A^ +2.32 (+0.34, +4.29)^A^ +0.54 (−0.34, +1.41)^A^ *0.105*	+0.14 (−1.41, +1.69)^A^ −0.33 (−1.41, +0.75)^A^ +0.70 (+0.23, +1.17)^A^ *0.217*	−1.16 (−2.39, +0.06)^B^ +0.51 (−0.31, +1.32)^A^ +0.40 (+0.04, +0.77)^A^ ***0.049***	−0.44 (−2.03, +1.15) ^AB^ +1.62 (+0.23, +3.01)^B^ −0.58 (−1.33, +0.18)^A^ ***0.028***

*In variables with 3 or more groups, each box will present letters A to C (superscript). It should be concluded that subgroups that share the same letters in the same box are non-significantly different.

y, years; M, months; BMI, body mass index; ProtU, protein/creatinine ratio in the urine; eGFR, estimated glomerular filtration rate.

*p* values are depicted in italics; statistically significant, *p* values (<.05) are shown in bold.

We found a non signficant trend when comparing pre-donation eGFR subgroups. The donors with lower eGFR pre-donation (<80 mL/min/1.73 m^2^) presented a negative eGFR change overall of −0.09 (95% CI, −0.63 to+0.44)mL/min/1.73 m^2^ vs. a positive shift in around 0.5 mL/min/1.73 m^2^ in the subgroups with ≥80 mL/min/1.73 m^2^ pre-donation (Table 4). Moreover, when analyzing eGFR variations by timespans, the subgroup with lower eGFR pre-donation presented a significant decline in eGFR in the period between 5 and 10 years of −1.16 (95% CI, −2.39 to +0.06) mL/min/1.73 m^2^/year, when compared to the other subgroups: +0.51 (95% CI, −0.31 to +1.32) mL/min/1.73 m^2^/year in the subgroup with pre-donation eGFR 80–90 mL/min/1.73 m^2^, vs. +0.40 (95% CI, +0.04 to +0.77) mL/min/1.73 m^2^/year in those with ≥90 mL/min/1.73 m^2^ pre-donation, *p* = 0.049. The lower function subgroup was associated with a more precocious increase in eGFR. The group with the highest eGFR pre-donation presented a more stable behavior, resembling the overall cohort, except for the last period of 10–15 years.

Pre-donation obesity was associated with a significantly greater decline of eGFR in the cohort over the entire period compared to normal weight donors (Table 4). Obese donors had a decrease of eGFR of −0.18 (95% CI −0.68 to +0.31) mL/min/1.73 m^2^/year, while the second group had an increase of +0.59 (95% CI +0.37, +0.80) mL/min/1.73 m^2^/year (*p* = 0.02). This difference was more apparent in the earlier periods post-donation (6 months–5 years), with varying directions. Initially, at 6 months to 2 years, obese donors experienced significantly higher decline of eGFR of −2.47 (95% CI, −4.92 to −0.03) mL/min/1.73 m^2^/year, *p* = 0.002. However, in the 2–5 year period, they showed a temporary recovery of eGFR of +1.18 (95% CI, −0.17 to +2.53) mL/min/1.73 m^2^/year.

**FIGURE 2:**
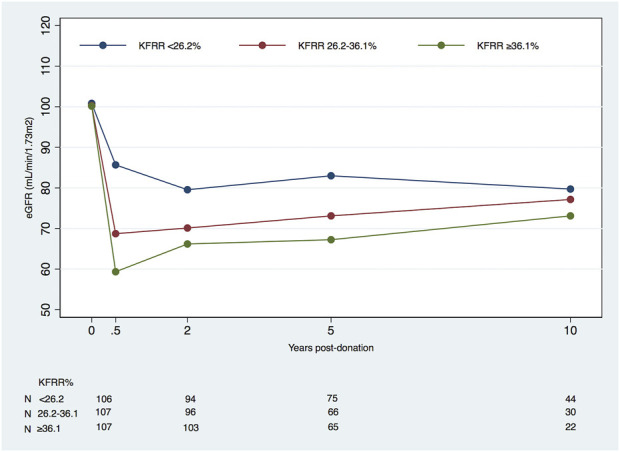
Mean eGFR (mL/min/1.73 m^2^) in living kidney donors by kidney function reduction rate (KFRR*) percentage subgroups at 6 months, starting at pre-donation and during post-donation follow-up (eGFR, estimated glomerular filtration rate). *KFRR post-donation = [−(eGFR6M-eGFRpre-donation)/eGFRpre-donation*100].

We carried out a separate analysis of %KFRR at 6 months post-donation, adjusted to the LD pre-donation factors (Table 5). The subgroup with a lower percentage of KFRR (<26.2%) had a significantly negative change in eGFR overall compared to the groups with higher loss rates of −0.21 (95% CI, −0.42 to +0.01) mL/min/1.73 m^2^/year vs. +0.53 (95% CI, +0.28 to +0.78) in the intermedium group and +0.65 (95% CI, +0.39 to +0.92) mL/min/1.73 m^2^/year in the group with KFRR >36.1%, *p* < 0.001. In the linear spline model, these differences only hold for 6 months to 2 years, where the three subgroups of kidney function recovery had significantly different eGFR changes (Figure 2).

**TABLE 5:** Changes in eGFR (mL/min/1.73 m^2^/year) in living kidney donors (n = 320) by % of KFRR post-donation donor group over different periods during follow-up from 6 months onward (multivariable analysis), adjusted to pre-donation variables previous analyzed: donor age, sex, BMI group: <25, 25–30, ≥30 kg/m^2^, diagnosis of hypertension, smoking habits, dyslipidemia, presence of proteinuria and eGFR group <80, 80–90 and ≥90 m/min/1.73 m^2^.

		Linear spline model			
	Overall	6M–2y	2y–5y	5y–10y	10–15y
%KFRR post-donation*,** <26.2 26.2–36.1 >36.1 *p*	−0.21 (−0.42, +0.01)^B^ +0.53 (+0.28, +0.78)^A^ +0.65 (+0.39, +0.92)^A^ ***<0.001***	−2.71 (−4.04, −1.39)^A^ +1.50 (+0.20, +2.80)^B^ +3.66 (+2.38, +4.94)^C^ ***<0.001***	+0.35 (−0.34, +1.03)^A^ +0.79 (+0.08, +1.51)^A^ +0.04 (−0.67, +0.76)^A^ *0.339*	+0.03 (−0.47, +0.53)^A^ +0.09 (−0.48, +0.66)^A^ +0.43 (−0.19, +1.05)^A^ *0.595*	−0.36 (−1.14, +0.42)^A^ −0.49 (−1.77, +0.80)^A^ −0.61 (−1.84, +0.61)^A^ *0.941*

*In variables with 3 or more groups, each box will present letters A to C (superscript). It should be concluded that subgroups that share the same letters in the same box are non-significantly different.

**KFRR post-donation = [−(eGFR6M-eGFRpre-donation)/eGFRpre-donation*100].

y, years; M, months; BMI, body mass index; ProtU, protein/creatinine ratio in the urine; eGFR, estimated glomerular filtration rate; KFRR, kidney function reduction rate post-donation.

*p* values are depicted in italics; statistically significant, *p* values (<.05) are shown in bold.

Table 6 presents the observed eGFR values in our cohort, categorized by different subgroups, before and after donation at 6 months, 2, 5, 10, and 15 years.

**TABLE 6:** Mean eGFR (mL/min per 1.73 m^2^) in living kidney donors by subgroup using the CKD-EPI equation, pre-donation, and post-donation at 6 months, 2 years, 5 and 10 years.

	Pre-donation	6M	2y	5y	10y
N	320	320	293	206	96
Overall	100.4 (14.6)	71.2 (16.3)	71.8 (14.9)	74.9 (16.1)	77.4 (14.8)
Age (years) <40 40–55 ≥ 55 *p*	112.1 (12.8)^A^ 99.1 (14.1)^B^ 91.8 (10.3)^C^ ***<0.001***	81.6 (15.8)^A^ 70.1 (15.7)^B^ 63.3 (12.3)^C^ ***<0.001***	81.7 (14.7)^A^ 70.7 (13.0)^B^ 65.1 (13.8)^C^ ***<0.001***	86.7 (13.2)^A^ 73.9 (14.8)^B^ 64.9 (13.6)^C^ ***<0.001***	83.9 (11.4)^A^ 77.7 (15.3)^A^ 68.3 (13.4)^B^ ***0.001***
Sex Male Female *p*	99.3 (14.3) 100.9 (14.8) *0.363*	70.3 (18.0) 71.6 (15.5) *0.596*	69.4 (15.9) 72.6 (14.4) *0.099*	73.5 (16.5) 75.4 (16.0) *0.459*	75.2 (17.5) 78.1 (13.9) *0.406*
BMI (kg/m^2^) <25 25–30 ≥30 *p*	102.4 (15.0)^A^ 99.0 (14.0)^A^ 97.2 (14.6)^A^ *0.060*	73.5 (16.8)^A^ 67.8 (13.8)^B^ 74.2 (20.6)^B^ ***0.007***	73.4 (15.7)^A^ 70.5 (13.3)^A^ 69.4 (16.2)^A^ *0.187*	78.4 (16.5)^A^ 70.5 (13.4)^B^ 75.0 (20.1)^A^ ***0.004***	78.7 (13.9)^A^ 76.0 (14.4)^A^ 80.7 (22.8)^A^ *0.583*
Hypertension No Yes *p*	101.7 (14.6) 93.6 (12.6) ***<0.001***	72.1 (16.4) 66.4 (15.0) ***0.022***	72.8 (14.9) 66.4 (13.9) ***0.007***	76.0 (16.2) 69.1 (14.1) ***0.025***	78.1 (14.6) 71.7 (15.7) *0.176*
Smoking habits No Yes *p*	99.3 (14.4) 106.5 (14.4) ***0.002***	70.3 (15.8) 76.6 (18.1) ***0.013***	71.4 (14.9) 74.1 (14.7) *0.256*	74.0 (16.0) 79.8 (16.0) *0.069*	77.0 (14.9) 79.7 (14.7) *0.552*
Dyslipidemia No Yes *p*	102.0 (13.8) 90.8 (16.0) ***<0.001***	72.6 (16.2) 62.7 (14.5) ***<0.001***	73.1 (14.7) 64.0 (13.7) ***<0.001***	76.3 (15.6) 65.1 (16.1) ***<0.001***	77.8 (14.4) 74.7 (18.0) *0.523*
ProtU 0.15–0.5 g/g No Yes *p*	100.0 (13.9) 101.5 (16.2) *0.375*	70.9 (15.7) 72.0 (17.6) *0.573*	71.5 (14.8) 72.2 (15.1) *0.714*	73.4 (15.0) 78.2 (18.0) *0.051*	77.0 (15.4) 79.5 (11.5) *0.546*
Predonation eGFR*(mL/min/1.73m^2^) <80 80–90 ≥90 *p*	71.6 (6.7)^A^ 85.7 (3.0)^B^ 106.8 (9.6)^C^ ***<0.001***	52.7 (10.8)^A^ 62.3 (12.1)^B^ 75.2 (15.3)^C^ ***<0.001***	56.1 (12.2)^A^ 63.6 (12.2)^A^ 75.3 (13.8)^B^ ***<0.001***	59.3 (17.7)^A^ 63.6 (12.5)^A^ 78.3 (14.8)^B^ ***<0.001***	60.5 (9.4)^A^ 71.8 (17.2)^AB^ 80.1 (13.4)^B^ ***<0.001***
%KFRR post-donation*,** <26.2 26.2–36.1 >36.1 *p*	100.8 (16.9)^A^ 100.3 (14.1)^A^ 100.2 (12.7)^A^ *0.941*	85.7 (16.1)^A^ 68.7 (10.5)^B^ 59.3 (8.6)^C^ ***<0.001***	79.6 (15.9)^A^ 70.1 (12.8)^B^ 66.2 (12.6)^B^ ***<0.001***	84.0 (16.8)^A^ 73.1 (14.1)^B^ 67.2 (12.7)^B^ ***<0.001***	79.7 (15.0)^A^ 77.2 (14.9)^A^ 73.1 (14.0)^A^ *0.234*

*In variables with 3 or more groups, each box will present letters A to C (superscript). It should be concluded that subgroups that share the same letters in the same box are non-significantly different.

**KFRR post-donation = [−(eGFR6M-eGFRpre-donation)/eGFRpre-donation*100].

Y, years; M, months; BMI, body mass index; ProtU, protein/creatinine ratio in the urine; eGFR, estimated glomerular filtration rate; FKRR, kidney function reduction rate post-donation; CKD-EPI, Chronic Kidney Disease-Epidemiology Collaboration equation.

*p* values are depicted in italics; statistically significant, *p* values (<.05) are shown in bold.

### Low eGFR and Proteinuria in LKD During Follow-Up

Table 7 depicts the eGFR category (ml/min/1.73 m^2^) for our cohort of LDs based on the last available SCr measurement. Notably, no donor reached eGFR <15 mL/min/1.73 m^2^, and only six reached CKD stages 3b and 4.

**TABLE 7:** eGFR (ml/mi/1.73 m^2^) category for donors based on the last available SCr measurement, using the CKD-EPI equation.

eGFR (ml/min/1.73 m^2^)	n = 320 n (%)
<15	0
15–30	2 (1)
30–45	4 (1)
45–60	55 (17)
60–90	205 (64)
≥90	54 (17)

Scr, serum creatinine; eGFR, estimated glomerular filtration rate; CKD-EPI, Chronic Kidney Disease-Epidemiology Collaboration equation.

We analyzed the prevalence of low eGFR by time frames after donation. We considered several cutoffs (Table 8). When we used eGFR <60 mL/min/1.73 m^2^, according to KDIGO definition of CKD [26], the prevalence of low eGFR during follow-up diminished from 25% at 6 months to 13% at 10 years. This was not an unexpected finding. We have observed a steady increase in eGFR from 6 months post-donation up to 10 years after donation. No donor had eGFR <30 mL/min/1.73 m^2^, and only one percent had eGFR <40 mL/min/1.73 m^2^. We selected the cutoff of 50 mL/min/1.73 m^2^, as we believe that it defines a more meaningful CKD status, to analyze the prevalence of low eGFR by subgroup over different periods during follow-up (Table 9). The prevalence of low eGFR remained overall low after 6 months, ≤5%. Older LDs (≥55 years) had a significantly higher prevalence of low eGFR from 6 months until 5 years of follow-up (13% for those ≥55 years vs. 3% for those 40–55 years and none in the younger group, *p* = 0.005), not thereafter. The same holds for donors with lower eGFR pre-donation. Those with higher KFRR (>36.1%) had a higher prevalence of lower eGFR at 6 months of 19% vs. 6% in the intermedium group and 0% in the group with KFRR <26.2%, *p* < 0.001. No difference in prevalence of lower eGFR was observed between KFRR subgroups thereafter.

**TABLE 8:** Prevalence of low eGFR (ml/min/1.73 m^2^) using the CKD-EPI equation, in living kidney donors during follow-up n (%).

	Predonation	6 M	2y	5y	10y
N	320	320	293	206	96
<60	0	79 (25)	68 (23)	38 (18)	12 (13)
<55	0	44 (14)	31 (11)	16 (8)	5 (5)
**<50**	**0**	**26 (8)**	**13 (4)**	**10 (5)**	**2 (2)**
<45	0	8 (3)	3 (1)	4 (2)	2 (2)
<40	0	3 (1)	2 (1)	2 (1)	1 (1)
<35	0	0	2 (1)	0	0
<30	0	0	0	0	0

M, months; y, years; CKD-EPI, Chronic Kidney Disease-Epidemiology Collaboration equation.

Bold is used to indicate the cutoff for defining a low estimated glomerular filtration rate in the cohort.

**TABLE 9:** Prevalence of eGFR<50 mL/min/1.73 m^2^, in living kidney donors by subgroup over different time periods during follow-up from 6 months onward n (%), using the CKD-EPI equation.

	Predonation	6M	2y	5y	10y
n	320	320	293	206	96
Overall	0	26 (8)	13 (4)	10 (5)	2 (2)
Age (years) <40 40–55 ≥ 55 *p*	—	1 (4) 14 (9) 11 (13) ***0.009***	1 (4) 4 (3) 8 (10) ***0.025***	0 3 (3) 7 (13) ***0.005***	0 1 (2) 1 (5) *0.446*
Sex Male Female *p*	—	9 (10) 17 (7) *0.515*	6 (7) 7 (3) *0.127*	3 (5) 7 (5) *0.729*	2 (8) 0 *0.061*
BMI (kg/m^2^) <25 25–30 ≥30 *p*	—	12 (8) 11 (8) 3 (9) *0.956*	8 (6) 2 (2) 3 (10) *0.063*	4 (4) 5 (6) 1 (5) *0.894*	1 (3) 0 1 (14) *0.064*
Hypertension No Yes *p*	—	18 (7) 8 (16) ***0.031***	9 (4) 4 (9) *0.138*	6 (3) 4 (12) *0.057*	1 (1) 1 (9) *0.217*
Smoking No Yes *p*	—	24 (9) 2 (4) *0.394*	13 (5) 0 *0.227*	10 (6) 0 *0.364*	2 (2) 0 *1*
Dyslipidemia No Yes *p*	—	17 (6) 9 (20) ***0.001***	7 (3) 6 (14) ***0.001***	6 (3) 4 (15) ***0.025***	1 (1) 1 (9) *0.217*
ProtU 0.15–0.5 g/g No Yes *p*	—	19 (8) 7 (7) *0.721*	9 (4) 4 (4) *1*	9 (6) 1 (2) *0.287*	2 (2) 0 *1*
Pre-donation eGFR (mL/min/1.73m^2^) <80 80–90 ≥90 *p*	—	14 (48) 9 (19) 3 (1) ***<0.001***	8 (31) 3 (7) 2 (1) ***<0.001***	5 (36) 3 (10) 2 (1) ***<0.001***	1 (14) 1 (7) 0 *0.051*
%KFRR post-donation <26.2 26.2–36.1 >36.1 *p*	—	0 6 (6) 20 (19) ***<0.001***	2 (2) 3 (3) 8 (8) *0.162*	2 (3) 3 (5) 5 (8) *0.395*	1 (2) 0 1 (5) *0.711*

*KFRR post-onation = [−(eGFR6M-eGFRpre-donation)/eGFRpre-donation*100].

y, years; M, months; BMI, body mass index; ProtU, protein/creatinine ratio in the urine; eGFR, estimated glomerular filtration rate; CKD-EPI, Chronic Kidney Disease-Epidemiology Collaboration equation; KFRR, kidney function reduction rate post-donation.

*p* values are depicted in italics; statistically significant, *p* values (<.05) are shown in bold.

These findings were accompanied by a non-significant rise in proteinuria after donation (Table 10). In fact, the prevalence of proteinuria decreased from 30% pre-donation to 10% 6 months after donation, and that prevalence remained stable afterward.

**TABLE 10:** Prevalence of proteinuria in living kidney donors pre-donation and over different periods during follow-up.

	Time				
	Pre-donation	6M	2y	5y	10y
n	302	283	251	172	78
Median (IQR)	0.11 (0.07–0.16)	0.08 (0.06–0.11)	0.07 (0.06–0.10)	0.08 (0.06–0.11)	0.07 (0.06–0.10)
≥0.15 g/g n (%)	96 (30)	30 (11)	19 (8)	18 (10)	6 (8)

n, number; %, percentage; IQR, interquartile range; g/g, protein g/creatinine g in a random urinary sample; M, months; Y, years.

## Discussion

In this cohort of 320 LDs, we found reassuring results about the evolution of long-term eGFR in LDs. Overall, the donors presented an average change in eGFR 6 months onward of +0.35 (95% CI, +0.20 to +0.50) mL/min/1.73 m^2^ per year. The period with the higher increase was from 6 months to 2 years with a mean increase of eGFR of +0.85 (95% CI +0.10 to +1.61) mL/min/1.73 m^2^ per year, and the recovery after donation plateaued at 10 years, after which the calculated mean change in eGFR is −0.24 (95% CI, −0.75 to +0.28) mL/min/1.73 m^2^ per year. To the best of our knowledge, this time span of 10–15 years post-donation trajectories have not been previously reported. As we hypothesized, when subgroups of donors were analyzed, we identified different kidney function recovery patterns. Obese LDs had a statistically significant overall worse recovery of eGFR compared to normal weight donors. The recovery trajectory of kidney function in obese donors showed a biphasic pattern at earlier timespans after donation, up to 5 years. The intermediate group of eGFR pre-donation has a better recovery than the extreme function groups. LDs with a lower %KFRR at 6 M (<26.2%) compared to eGFR pre-donation presented a significantly higher decrease of eGFR in the overall period compared to the other two groups. Still, the differences between groups only hold for the time frame of 6 M to 2 years. Moreover, an eGFR <50 mL/min/1.73 m^2^ was a rare event, and the proteinuria prevalence did not increase during the follow-up.

A substantial nephron loss is expected in the aging kidneys [27]. Renal function decline was well-characterized in several general and healthy populations [28–30]. Studies in the healthy Swedish population have demonstrated that the mean decline in GFR was 4 mL/min/1.73 m^2^ per decade up to 50 years of age and then decreased annually by 1 mL/min [29, 30]. In a large series of healthy potential LDs, Fenton et al. [28] found the measured GFR (mGFR) had a linear decline after 35 years of 6.6–7.7 mL/min/1.73 m^2^/decade. In the long term (10–15Y), our cohort’s mean change in eGFR stayed below these references.

Increases in GFR long-term after donation have been described for years [31, 32], but most studies lack detailed data about GFR trajectories. Matas et al. [32] found that the increase in eGFR continued until at least 20 years post-donation in their study of 2002 predominantly white donors.

Kasiske et al. [14], in a prospective observational study, compared 205 living donor candidates and 203 healthy controls with serially measured iohexol GFR. Between 6 M and 9 years, the mean change in mGFR was significantly different among donors +0.02 (95% CI, −0.16 to −20) mL/min/1.73 m^2^/year vs. −1.26 (95% CI, −1.52 to −1.00) mL/min/1.73 m^2^/year in controls. Lam et al. [10], in a retrospective cohort study of LDs in Alberta, in 2002–2016, matched 604 donors to 2,414 healthy non-donors from the general population. Overall, from 6 weeks onwards, the eGFR increased by 0.35 mL/min/1.73 m^2^ per year (95% CI, +0.21 to +0.48) in donors and significantly decreased by −0.85 mL/min/1.73 m^2^ per year (95% CI, −0.94 to −0.75) in the matched non-donors [10]. Our data is largely in line with these observations.

After nephrectomy, there is compensatory hyperfiltration in the remaining kidney, such that while a donor immediately loses approximately 50% of the kidney mass, the net reduction in GFR early after the donation is only approximately 30% [16]. The mechanisms of compensatory hyperfiltration are not clear yet. In a remarkable long-term study of glomerular hemodynamics after kidney donation [12], it was noted that adaptive hyperfiltration after donor nephrectomy was attributable to hyperperfusion and hypertrophy of the remaining glomeruli, without glomerular hypertension in most donors, and these changes were sustained throughout the late post-donation period, without significant albuminuria [12]. Nevertheless, there is concern that adaptive hyperfiltration might result in faster progression of kidney disease in certain groups of donors with less functional reserve, such as those older, obese, or hypertensive [12].

Van de Weijden et al. [33], in a cohort of 1024 donors, found that individuals with a more pronounced increase in single-kidney GFR at 3 months after donation had better long-term kidney function, independent of pre-donation GFR and age. The authors hypothesized that an early increase in eGFR may reflect a more physiologic adaptation mechanism to an acute reduction in renal mass and a better renal functional reserve. These results could help personalize LD follow-up [33].

Our results were surprising when we evaluated the impact of the percentage of KFRR at 6M in the trajectories of eGFR over time. The individuals with less KFRR (<26.2%) in the first 6 months had a significantly higher decrease of eGFR in the overall period compared to the other subgroups. However, the significant differences between the three subgroups only held from 6 months to 2 years. Distinctly, it was not a predictive factor of long-term renal function in our cohort, and we could not support the hypothesis of Weijden et al. [33].

Lower pre-donation eGFR has been associated with lower post-donation eGFR and a higher risk of ESRD [34, 35]. Tan et al. [11], in a retrospective cohort of 174 Southeast Asian LKDs, described that pre-nephrectomy eGFR was a good predictor of post-donation eGFR, especially in the short term (<6 M). Still, it was limited to <5 years and did not necessarily translate into a long-term (>5 Y) reduction in post-donation eGFR. In our cohort, we could not correlate better pre-donation eGFR with improved recovery of post-donation eGFR. We could hypothesize, as studied by Chakkera et al. [36], that adaptation reserves for increasing filtration after nephrectomy may be limited in donors with a high eGFR.

Several studies reported worse outcomes in obese LDs, including an increased risk of ESRD [8, 9, 34], although it is not a contraindication for donation [16]. Ibrahim et al. [35], in a white LDs population, showed that each increase of 1 unit in BMI pre-donation was associated with a 3%–10% higher risk of proteinuria and reduced GFR. In our study, obese donors at the time of donation experienced significantly worse overall eGFR change from 6 months onward compared to normal-weight donors. However, these differences only hold for the initial time frames (6 M–5 Y). Some of these donors, with preexisting obesity-related hyperfiltration, may have a diminished capacity to undergo further adaptive hyperfiltration after nephrectomy compared to a normal-weight donor [37]. Our cohort results clearly red-flagged this population and deserve further investigation concerning the mechanisms involved and potential preventive primary or secondary measures that might be indicated [38].

In our cohort, we did not find significantly different trajectories of eGFR when considering donor age, sex, hypertension, dyslipidemia, and the presence of proteinuria pre-donation. The aging healthy kidney is associated with lower renal function and blunted adaptative capacity [30]. Our cohort of older donors has not been associated with worse outcomes for their recipients [21]. A comprehensive evaluation of an older LD could be a good strategy for many LDs pairs.

LDs diagnosed with hypertension pre-donation did not present a distinctive slope of eGFR after donation. Hypertension represents a leading cause of cardiovascular morbidity and mortality. It is associated with CKD and the risk of ESRD in the general population and is a frequently reported cause of ESRD in living donors [39]. Furthermore, it can reduce the renal reserve and limit the expected post-donation compensation [40]. Our results could be explained by our thorough practice in selecting these donors. Sanchez et al. [23], in a population of LDs, found that the risks for the different clinical outcomes, including eGFR < 60, 45, or 30 mL/min/1.72 m^2^ or ESRD, between those with and without hypertension at the time of donation were not different. A different issue, the effect of hypertension after donation on the eGFR trajectories, is beyond the scope of this work.

The proportion of LDs with low eGFR, defined as eGFR <50 mL/min/1.73 m^2^ was overall small, 5% or less after the 6 months, decreased with the follow-up time, which is expected with progressive improvement in kidney function, suggesting that the decline in eGFR was not progressive in the majority of LDs. The results on the prevalence of proteinuria in the follow-up period support this theory, pointing away from the hypothesis of hyperfiltration after a donation from the remnant nephron [38], which focused on the role of glomerular hypertension in the remaining nephrons as the main pathway for progressive renal damage and consequent glomerular leakage of proteins.

Our study has several limitations. First, donors were evaluated retrospectively, and unobserved confounders may have introduced bias. Second, we have not assessed a non-donor control group, although we can compare our results with studies of the evolution of eGFR with aging in healthy European populations [28–30]. Third, our cohort consisted almost exclusively of Caucasian patients, limiting the generalization of our results. In addition, eGFR using estimation equations to assess kidney function has limitations, but it is the common practice in most transplant centers and agrees with the International Guidelines [16]. In addition, an added value of our study cohort is its larger size and the availability of serial SCr measurements. Nevertheless, longer follow-up studies must be required; prospective studies are necessary to allow a cause-effect analysis of the parameters studied. Furthermore, the influence of *de novo* comorbidities such as hypertension and diabetes were not evaluated as modifiers of the evolution of kidney function after donation.

## Conclusion

Our data show that eGFR post-donation recovery is significant and may last until 10 years post-donation. Moreover, an eGFR <50 mL/min1.73 m^2^ was a rare observation, having a prevalence of 5% or less in the 2–15 years span. These observations confirm that in a carefully selected cohort of donors, the occurrence of a significant kidney function loss or accelerated decline is exceptional. However, some subgroups of donors presented a more ominous kidney function trajectory pattern, pointing to the necessity of tailored follow-up.

## Data Availability

The raw data supporting the conclusions of this article will be made available by the authors, without undue reservation.
