# Supplementary material for: Longitudinal Trajectories of Estimated Glomerular Filtration Rate in a European Population of Living Kidney Donors
Source: Transpl Int. 2024 Aug 26;37:13356. doi: 10.3389/ti.2024.13356 (PMC11381247; doi:10.3389/ti.2024.13356)
Supplement: Supplementary file 1 [file Table1.DOCX]

Supplementary Table 1. Comparison of baseline characteristics of the cohort study with living donors not included due to missing inclusion criteria.

|  | n=320 | n=44 | *p* |
| --- | --- | --- | --- |
| Age (years), Mean±SD | 47.3±10.5 | 42.4±11.3 | 0.004 |
| Age (years), n (%)  < 40  40-55  >= 55 | 81 (25)  154 (48)  85 (27) | 17 (38)  22 (49)  6 (13) | 0.08 |
| Sex F:M, n (%) | 227 (71):93(29) | 20 (44):25(56) | 0.037 |
| BMI kg/m^2^, Mean±SD | 25.3±3.3 | 24.4±2.9 | 0.072 |
| BMI kg/m^2^, n (%)  <25  25-30  >=30 | 155 (48)  132 (41)  33 (10) | 30 (67)  13(29)  2(4) | 0.074 |
| Smoking habits, n (%) | 48 (15) | 13(29) | 0.019 |
| Hypertension, n (%) | 51 (16) | 4(9) | 0.27 |
| Dyslipidemia, n (%) | 44 (14) | 4(9) | 0.483 |
| ProtU 0.15-0.5 g/g, n (%) | 96 (30) | 4 (9) | 0.002 |
| Pre-donation SCr mg/dL, Mean ± SD | 0.75±0.16 | 0.8±0.15 | 0.025 |
| Pre- donation eGFR mL/min/1.73m^2^, Mean ± SD | 100.4±14.6 | 101.5±15.5 | 0.636 |
| Pre- donation eGFR mL/min/1.73m^2^, n (%)  <80  80-90  >=90 | 29 (9)  48 (15)  243 (76) | 4 (9)  6 (13)  35 (8) | 1 |

SD - standard deviation; n - number; F - female: M - male; BMI - Body Mass Index; ProtU - protein/creatinine ratio in the urine; ProtU 0.15-0.5 g/g- a ratio protein/creatinine of 0.15 to 0.5 g/g in a urinary sample; SCr - Serum creatinine; eGFR - estimated glomerular filtration rate, IQR - interquartile range;

The living donors’ population non-included in the study cohort included a younger cohort, with more males, more smoking habits, and less prevalence of proteinuria

Supplementary Table 2. Estimated glomerular filtration rate (ml/min/1.73 m^2^) category for donors non-included in the study based on the last available SCr measurement, using the CKD-EPI equation

| eGFR (ml/min/1.73 m^2^) | n=38*  n (%) |
| --- | --- |
| <15 | 0 |
| 15-30 | 0 |
| 30-45 | 2(5.2) |
| 45-60 | 5 (13.2) |
| 60-90 | 24 (63.2) |
| >=90 | 7 (18.4) |

Scr- serum creatinine: eGFR- estimated glomerular filtration rate; CKD-EPI- Chronic Kidney Disease-Epidemiology Collaboration equation

*6 living donors don’t have any available serum creatinine after the donation
